# Description of a new genus and species for a common and widespread Amazonian satyrine butterfly (Lepidoptera: Nymphalidae: Satyrinae: Satyrini)

**DOI:** 10.7717/peerj.10324

**Published:** 2020-12-04

**Authors:** Shinichi Nakahara, Gerardo Lamas, Keith Willmott, Marianne Espeland

**Affiliations:** 1 McGuire Center for Lepidoptera and Biodiversity, Florida Museum of Natural History, University of Florida, Gainesville, FL, United States of America; 2 Department of Entomology and Nematology, University of Florida, Gainesville, FL, United States of America; 3 Museo de Historia Natural, Universidad Nacional Mayor de San Marcos, Lima, Peru; 4 Arthropoda Department, Zoological Research Museum Alexander Koenig, Bonn, Germany

**Keywords:** Euptychiina, Monotypic taxa, Systematics, Taxonomy

## Abstract

We here propose a new monotypic butterfly genus *Scriptor* Nakahara & Espeland, **n. gen.** to accommodate a new species, *S. sphenophorus* Lamas & Nakahara, **n. sp.**, described and named herein. *Scriptor sphenophorus* **n. gen. and n. sp.**is a relatively common and widespread butterfly species which is recovered as a member of the so-called “*Splendeuptychia* clade” in the nymphalid subtribe Euptychiina, based on our molecular phylogenetic analysis using a maximum likelihood approach. Nevertheless, its sister group is not confidently resolved in any analysis, supporting a relatively distant relationship to any described genus as well as our decision to establish a new monotypic genus. We further discuss the proposed taxonomy in the light of frequent criticism of the description of monotypic taxa, as well as emphasize the importance of incorporating multiple evidence when describing new genera, illustrated by reference to several recent generic descriptions in this subtribe.

## Introduction

It hardly needs saying that taxonomic hypotheses should be generated by incorporating multiple layers of evidence, to reduce the likelihood of creating invalid names or of those names not being broadly accepted by user communities. In proposing a new generic name, two critical pieces of information should accompany a genus description: (1) support for monophyly of the new genus; (2) evidence that establishment of the new genus does not result in non-monophyly of an existing genus. Despite recent arguments by Páll-Gergely (2017), molecular data often provide critical supporting evidence to assess generic monophyly, especially in groups with minimal or conflicting morphological characters. At a time when genetic data is used extensively in systematics, including support from such data must become a standard component of generic descriptions to avoid creating non-monophyletic genera.

The focal group of this study, the largely Neotropical satyrine subtribe Euptychiina, provides a good example of the need for sound phylogenetic studies to support a stable generic classification. This group contains many polyphyletic and paraphyletic genera, resulting mainly from a historical study by Forster (1964) that was conducted without phylogenetic analysis of any kind. Nevertheless, despite the widespread acceptance of the importance of phylogenetic analyses to support generic descriptions, some recent publications describing Euptychiina genera have not used such analyses (Costa et al., 2016; Andrade et al., 2019). A majority of studies in Euptychiina, however, have emphasized the need for comprehensive phylogenetic analyses, and a number of recent papers have provided descriptions of new genera in order to maintain the monophyly of existing genera (e.g.,  Nakahara et al., 2016; Nakahara et al., 2019a; Willmott et al., 2019).

*Magneuptychia* (Forster, 1964) is a species-rich euptychiine genus that has been shown to be polyphyletic in a number of molecular studies (e.g., Espeland et al., 2019), although recent attempts to revise its systematics did not incorporate phylogenetic analyses (Costa et al., 2016; Andrade et al., 2019). A study is therefore underway by the authors and collaborators to fully revise *Magneuptychia*, through a comprehensive analysis of Euptychiina (Espeland et al. in prep.), as well as papers focused on individual clades (e.g., Nakahara et al. in review). Apart from being polyphyletic, one of the major issues concerning *Magneuptychia* is the species-level classification of the “*Magneuptychia fugitiva* species group” and the “*Magneuptychia ocypete* species group”, where morphological homogeneity coupled with infra-specific and inter-specific variability makes species delimitation particularly challenging (Benmesbah et al., 2018; Zacca et al., 2017). Here we focus on a common and widespread Amazonian species long thought to be related to one of these two groups in “*Magneuptychia*”. The species was listed as an undescribed species in *Magneuptychia* by Lamas (2004), and a long series of specimens housed at various museums are curated under this genus. Nevertheless, phylogenetic analysis of molecular data suggest that this common Amazonian species is relatively isolated and warrants a new generic status.

## Material and Methods

**Museum visits and field work.** Specimens relevant to this study were examined at the following public and private collections: **BME** - Bohart Museum of Entomology, University of California Davis, Davis, USA; **CMNH** -Carnegie Museum of Natural History, Pittsburgh, USA; **DZUP** - Entomological Collection Padre Jesus Santiago Moure, Departamento de Zoologia, Universidade Federal do Paraná, Curitiba, Brazil; **FLMNH** - McGuire Center for Lepidoptera and Biodiversity, Florida Museum of Natural History, University of Florida, Gainesville, USA; **MIPE** - Mike J. Perceval collection, Surrey, UK; **MUSM** - Museo de Historia Natural, Universidad Nacional Mayor de San Marcos, Lima, Peru; **PUCE** - Pontificia Universidad Católica del Ecuador, Quito, Ecuador; **ZSM** - Zoologische Staatssammlung München, Munich, Germany; **ZUEC** - Museu de Zoologia ‘Adão José Cardoso’, Universidade Estadual de Campinas, Campinas, Brazil. Additional specimens were obtained during the course of field surveys by the authors as part of long-term research projects aimed to study the butterfly fauna of various South American countries. The necessary field permits were arranged by the Instituto Nacional de Biodiversidad (Quito, Ecuador) and the Ecuadorian Ministerio del Ambiente, most recently under the project ‘Diversity and Biology of Lepidoptera in Ecuador’ (No. 006-19 IC-FLO-FAU-DNB/MA).

**Table 1:** A list of primers and PCR reaction conditions relevant to this study.

**Sequence 5′-3′**	**Gene**	**Primer_name**	**Annealing temp. (**°**C)**	**Direction**	**References**
CCAGGATWTTTAATTGGDGATGA	**COI (6 part)**	COI_bc_EuF2	51	Forward	Nakahara et al. (2019c)
GGATTTGGWAATTGATTARTYCC	**COI (6 part)**	COI_bc_EuF3	51	Forward	Nakahara et al. (2019c)
AGTATYGTAGAAAATGGAGCTGG	**COI (6 part)**	COI_bc_EuF4	56	Forward	Nakahara et al. (2019c)
TTTGAGCTGTHGGAATTACAGC	**COI (6 part)**	COI_bc_EuF6	56	Forward	Nakahara et al. (2019c)
TATTATTTATACGVGGRAAAGCTA	**COI (6 part)**	COI_bc_EuR2X	51	Reverse	Nakahara et al. (2019c)
GTAATAGCTCCRGCTAAAACAG	**COI (6 part)**	COI_bc_EuR5X	51	Reverse	Nakahara et al. (2019c)
AAAAATTATAATAAAAGCATGRGC	**COI (6 part)**	COI_bc_TegR1	51	Reverse	Nakahara et al. (2019c)
ATTGTRGTAATAAAATTAATAGCTCC	**COI (6 part)**	COI_bc_TegR4X	56	Reverse	Nakahara et al. (2019c)
TAAACTTCAGGATGACCAAAAA	**COI (1 or 6 part)**	HCO_nym	56 (45 for 1 and 2 part)	Reverse	Nakahara et al. (2015)
WGGGGGGTAAACTGTTCATCC	**COI (2 part)**	K699	56 (45 for 2 part)	Reverse	Elias et al. (2007)
TTTCTACAAATCATAAAGATATTGG	**COI (1, 2 or 6 part)**	LCO_nym	56 (45 for 1 and 2 part)	Forward	Nakahara et al. (2015)
CCTGGTAAAATTAAAATATAAACTTC	**COI (2 part)**	Nancy	45	Reverse	Monteiro & Pierce (2001)
GGATCACCTGATATAGCATTCCC	**COI (2 part)**	Ron	45	Forward	Monteiro & Pierce (2001)
GCYGARCGYGARCGTGGTATYAC	**Ef1a (1 or 3 part)**	ef44	58	Forward	Monteiro & Pierce (2001)
ACAGCVACKGTYTGYCTCATRTC	**Ef1a**	efrcM4	58	Reverse	Monteiro & Pierce (2001)
CATRTTGTCKCCGTGCCARCC	**Ef1a (3 part)**	Monica	58	Reverse	Monteiro & Pierce (2001)
AARGCTGGRGCTGAATATGT	**GAPDH**	HybFrigga	46 (62, - 1 per cycle for the first 16 cycles)	Forward	Wahlberg & Wheat (2008)
GWTTGAATGTACTTGATRAGRTC	**GAPDH**	HybBurre	46 (same as above)	Reverse	Wahlberg & Wheat (2008)
YGCTCAYTTGGAWGGHGGGC	**GAPDH (2 part)**	GAPDH 42F	46 (same as above)	Forward	This study
WACWGGYACACGGAAWGCCA	**GAPDH (2 part)**	GAPDH 426R	46 (same as above)	Reverse	This study
YAACTTTGAARTATTGAAGGY	**GAPDH (2 part)**	GAPDH 213F	46 (same as above)	Forward	This study
ATGACACGGCTVGARTARGCA	**GAPDH (2 part)**	GAPDH 690R	46 (same as above)	Reverse	This study
ATGGCNGARGARAAYTGGAAYGA	**RpS5**	rpS5degF	46 (same as above)	Forward	Wahlberg & Wheat (2008)
CGGTTRGAYTTRGCAACACG	**RpS5**	rpS5degR	46 (same as above)	Reverse	Wahlberg & Wheat (2008)
GCWGACATTCCDGARATCAAR	**RpS5 (2 part)**	rps5_56F	46 (same as above)	Forward	This study
RCGDACDGCCATHARTTTCTTRCC	**RpS5 (2 part)**	rps5_295R	46 (same as above)	Reverse	This study
ATGATGCAYGGAAGAAACAA	**RpS5 (2 part)**	rps5_251F	46 (same as above)	Forward	This study
GATGAACCCTTRGCAGCATT	**RpS5 (2 part)**	rps5_576R	46 (same as above)	Reverse	This study

**Table 2:** GenBank Accession number information for DNA sequences used in this study.

**Voucher code**	**Genus**	**Species**	**COI**	**Ef1a**	**RpS5**	**GAPDH**	**Locality (decimal latitude and longitude)**
CP01-19	*Splendeuptychia*	*ashna*	GU205865	GU205921	GU206040	GU205979	Peru: Madre de Dios: Tambopata Research Center (−13.15, −69.617)
KW-080512-02	*Paryphthimoides*	*jorupe*	MF084829	MT787268	MF084839	MF084837	Ecuador: Loja: Reserva Jorupe, W Macará (−4.379, −79.904)
NW108-6	*Cissia*	*myncea*	DQ338581	DQ338933	GQ357556	GQ357427	Brazil: São Paulo: Picinguaba (−23.367, −44.833)
LEP-19673	*Magneuptychia*	*fugitiva*	MG010693	MT787275	MT787264	MT787284	French Guiana: St-Laurent du Maroni: St. Jean du Maroni (5.4, −54.083)
KW-140622-02	*Magneuptychia*	*alcinoe*	MT787245	MT787269	N/A	N/A	Ecuador: Zamora-Chinchipe: Quebrada de los Rubies (−4.877, −79.209)
LEP-17603	*Yphthimoides*	*maepius*	MT787253	MT787274	MT787263	MT787283	Ecuador: Morona-Santiago: km 47.6 Santiago-Puerto Morona rd. (−2.937, −77.747)
LEP-34315	*Magneuptychia*	*opima*	MT787254	MT787276	MT787265	MT787285	Ecuador: Pastaza: Yutsuntsa (−2.351, −76.454)
KW-081111-35	*Magneuptychia*	*louisammour*	MT787243	MT787267	MT787258	MT787278	Ecuador: Orellana: Boca del Río Añangu (−0.529, −76.395)
CP01-32	*Magneuptychia*	*louisammour*	GU205848	GU205904	GU206020	GU205961	Peru: Madre de Dios: Tambopata Research Center (−13.15, −69.617)
CP02-41	*Magneuptychia*	*ocypete*	GU205849	GU205905	GU206021	GU205962	Peru: Madre de Dios: Tambopata Research Center (−13.15, −69.617)
BC-DZ Willmott-188	*Scriptor*	*sphenophorus*	MT787244	N/A	N/A	N/A	Brazil: Acre: Parque Nacional Serra do Divisor, Porção Norte (−7.442, −73.659)
MGCL-LOAN-072	*Scriptor*	*sphenophorus*	MT787255	N/A	N/A	N/A	Brazil: Pará: [Rio] Tapájos (−4.268, −55.985)
MGCL-LOAN-319	*Scriptor*	*sphenophorus*	MT787256	N/A	N/A	N/A	Brazil: Pará: [Rio] Tapájos (−4.268, −55.985)
LEP-17572	*Scriptor*	*sphenophorus*	N/A	MT787273	MT787262	MT787282	Ecuador: Morona-Santiago: km 47.6 Santiago-Puerto Morona rd. (−2.937, −77.747)
LEP-08957	*Scriptor*	*sphenophorus*	MT787246	N/A	N/A	N/A	Ecuador: Pastaza: Kapawi Lodge (−2.542, −76.859)
DNA99-051	*Scriptor*	*sphenophorus*	AY508555	AY509081	N/A	N/A	Ecuador: Napo: no specific locality
LEP-10403	*Scriptor*	*sphenophorus*	MT787249	N/A	N/A	N/A	Ecuador: Sucumbíos: Cuyabeno Lodge, across lagoon (−0.005, −76.173)
LEP-15058	*Scriptor*	*sphenophorus*	MT787250	N/A	N/A	N/A	Ecuador: Orellana: Estación Científica Yasuní, parcela 50 Ha (−0.682, −76.4)
LEP-15060	*Scriptor*	*sphenophorus*	MT787251	N/A	N/A	N/A	Ecuador: Orellana: Estación Científica Yasuní (−0.674, −76.397)
LEP-15062	*Scriptor*	*sphenophorus*	MT787252	N/A	N/A	N/A	Ecuador: Orellana: Estación Científica Yasuní (−0.674, −76.397)
LEP-09792	*Euptychoides*	*nossis*	MT787247	MT787271	MT787260	MT787280	Ecuador: El Oro: Buenaventura, Río Moro Moro (−3.638, −79.747)
KW-140716-05	*Euptychoides*	*nossis*	MT787241	MT787270	MT787259	MT787279	Ecuador: Carchi: Santa Rosa (0.827, −78.128)
LEP-10058	*Euptychoides*	*nossis*	MT787248	MT787272	MT787261	MT787281	Ecuador: Carchi: Chical ’primera cordillera’ (0.929, −78.178)
LEP-15102	*Colombeia*	*mycalesis*	MT787242	MT787266	MT787257	MT787277	Ecuador: Esmeraldas: Finca Cypris (1.011, −78.609)
CP04-51	*Euptychoides*	*hotchkissi*	GU205836	GU205892	GU206009	GU205949	Peru: Junín: 1 km S Mina Pichita (−11.088, −75.418)

**Morphological study.** We used standard entomological techniques to examine the morphology of the specimens used in this study. Abdomens were removed and soaked in hot 10% potassium hydroxide 10% for 5–10 min and then dissected. They were subsequently stored in glass tubes and/or small plastic vials filled with glycerin. Wing venation was visualized by clearing scales of the ventral surface using 70% ethanol. These morphological features were studied and drawn using a Leica MZ 16 stereomicroscope at different magnifications up to 100x. The terminology associated with wings and genitalia largely follows Miller (1970: 44), Peña & Lamas (2005) and Klots (1956) (but see Nakahara et al., 2018a; Nakahara et al., 2018b for some modifications), and immature stage terminology follows Stehr (1987) and Cosmo, Barbosa & Freitas (2014). We use the following abbreviations throughout the text: **DFW**: dorsal forewing; **DHW**: dorsal hindwing; **VFW**: ventral forewing; **VHW**: ventral hindwing.

**Molecular work.** Methods for DNA extractions, design of internal primers, polymerase chain reaction (PCR) and Sanger sequencing parameters for the first half of the mitochondrial gene *cytochrome oxidase I* (COI) (commonly known as the ‘DNA barcode’ (Hebert et al., 2003)) followed Nakahara et al. (2019b). Additionally, three nuclear gene sequences, namely *elongation factor 1 alpha* (Ef1a), *glyceraldhyde-3-phosphate dehydrogenase* (GAPDH) and *ribosomal protein S5* (Rps5), were also obtained as described by Nakahara et al. (2018b). All primers and PCR reaction conditions used to amplify these four genes are listed in Table 1. These four genes were amplified for the selected taxa in the so-called “*Splendeuptychia* clade” *sensu* Peña et al. (2010), as well as representatives throughout the Euptychiina, and were used to infer the phylogeny to test our taxonomic hypothesis. The total dataset included 25 samples and 3,177 base pairs. Information about the sequences used in this study, including new DNA sequence data generated as part of this study, are provided in Table 2 with GenBank voucher codes. We performed a phylogenetic analysis with maximum likelihood as the optimality criterion, based on the concatenated dataset of the aforementioned genes. The phylogenetic analyses using IQ-TREE v2.0.5 (Minh et al., 2020) were largely performed as described by Nakahara et al. (2019a), with some notable differences, including data partitioned to codon position and application of best-fit substitution models individually derived through ModelFinder (Kalyaanamoorthy et al., 2017) (Table 3). We ran ten independent analyses based on our concatenated dataset in total, and calculated branch supports through 2,000 replications of both ultrafast bootstrap (UFBoot) (Hoang et al., 2018) with the “-bnni” option to reduce model violation, coupled with the Shimodaira-Hasegawa-like approximate likelihood ratio test (SH-aLRT) (Guindon et al., 2010). The following commands were used to run these analyses in IQ-TREE v2.0.5: iqtree2 -s infile.phy -p part_codon.txt -m scheme.nex -nt AUTO -pre Run_10runs -B 2000 -bnni -alrt 2000 –runs 10. The tree with the highest likelihood score for the above dataset was rooted with *Splendeuptychia ashna* (Hewitson, 1869) based on previous results (Espeland et al., 2019). We here use the generic names *Cissia* Doubleday, 1848 and *Magneuptychia* for taxa associated with this genus mainly in Lamas (2004), in addition to *Cissia maripa* Brévignon, 2005, although work is underway to provide an appropriate classification for these taxa in other genera.

**Table 3:** Best-fit substitution models by partition derived from ModelFinder and applied in this study.

**Codon position**	**Model**
COI 1st	TIM3+F+G4
COI 2nd	HKY+F+I
COI 3rd	K3Pu+F+G4
EF1a 1st	F81+F+I
EF1a 2nd	F81+F+I
EF1a 3rd	HKY+F+G4
GAPDH 1st	F81+F
GAPDH 2nd	F81+F+I
GAPDH 3rd	TN+F+G4
RPS5 1st	TIM2e
RPS5 2nd	JC+I
RPS5 3rd	TNe

**Nomenclatural acts**. The electronic version of this article in portable document format will represent a published work according to the International Commission on Zoological Nomenclature (ICZN), and hence the new names contained in the electronic version are effectively published under that Code from the electronic edition alone (see Articles 8.5–8.6 of the Code). This published work and the nomenclatural acts it contains have been registered in ZooBank, the online registration system for the ICZN. The ZooBank Life Science Identifiers (LSIDs) can be resolved and the associated information can be viewed through any standard web browser by appending the LSID to the prefix http://zoobank.org/. The LSID for this publication is as follows: urn:lsid:zoobank.org:pub:DC9E9E4F-0822-4A0A-BAFB-16F078089C47. The online version of this work is archived and available from the following digital repositories: PeerJ, PubMed Central, and CLOCKSS.

## Results

**Table:** 

**Figure 1:**
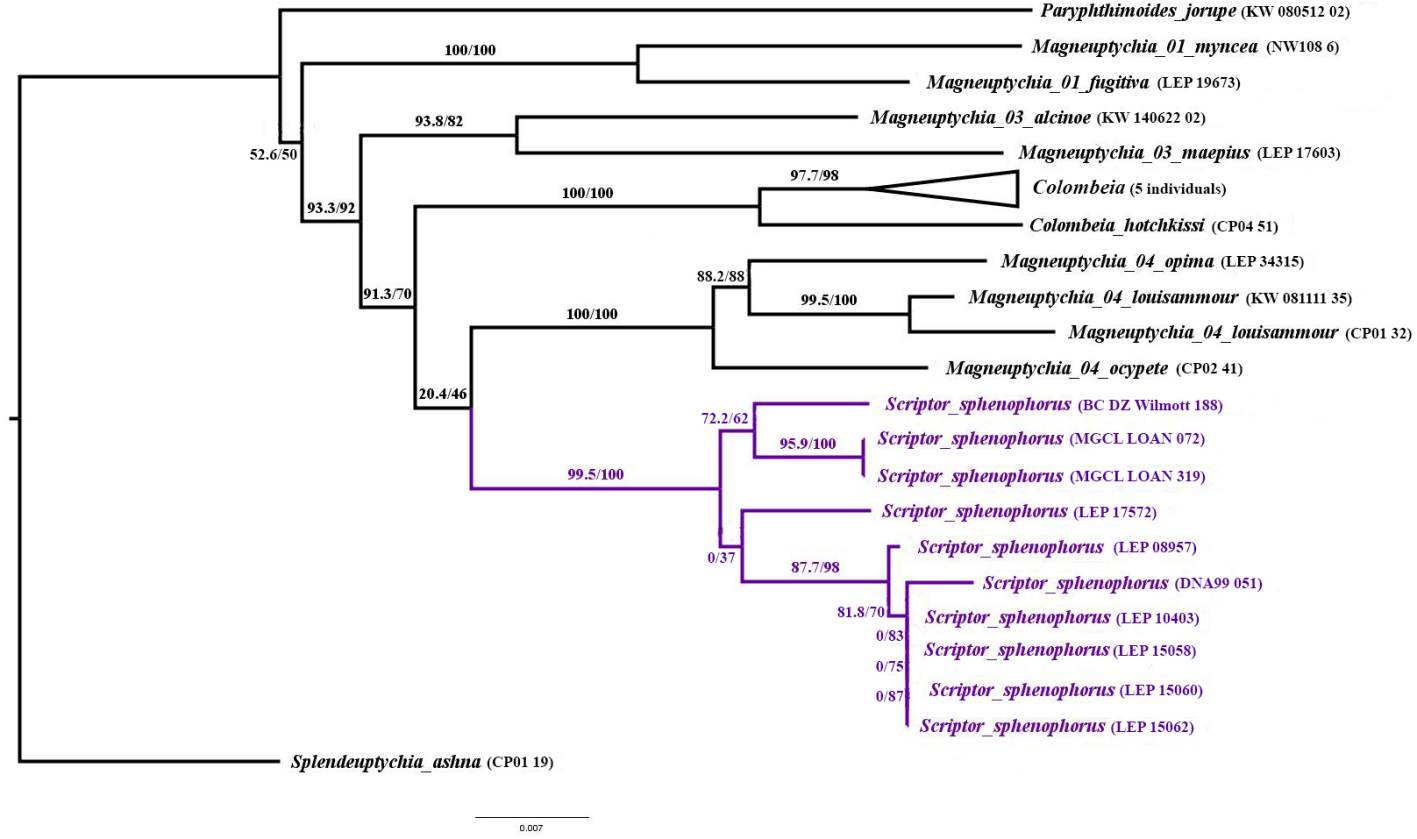
Maximum likelihood tree (LnL = − 8762.690) inferred in IQ-TREE v2.0.5, showing the low support for relationships among *Scriptor***n. gen.** and related taxa in the “*Splendeuptychia* clade”. Numbers beside branches are SH-aLRT/UFBoot values.

**Figure 2:**
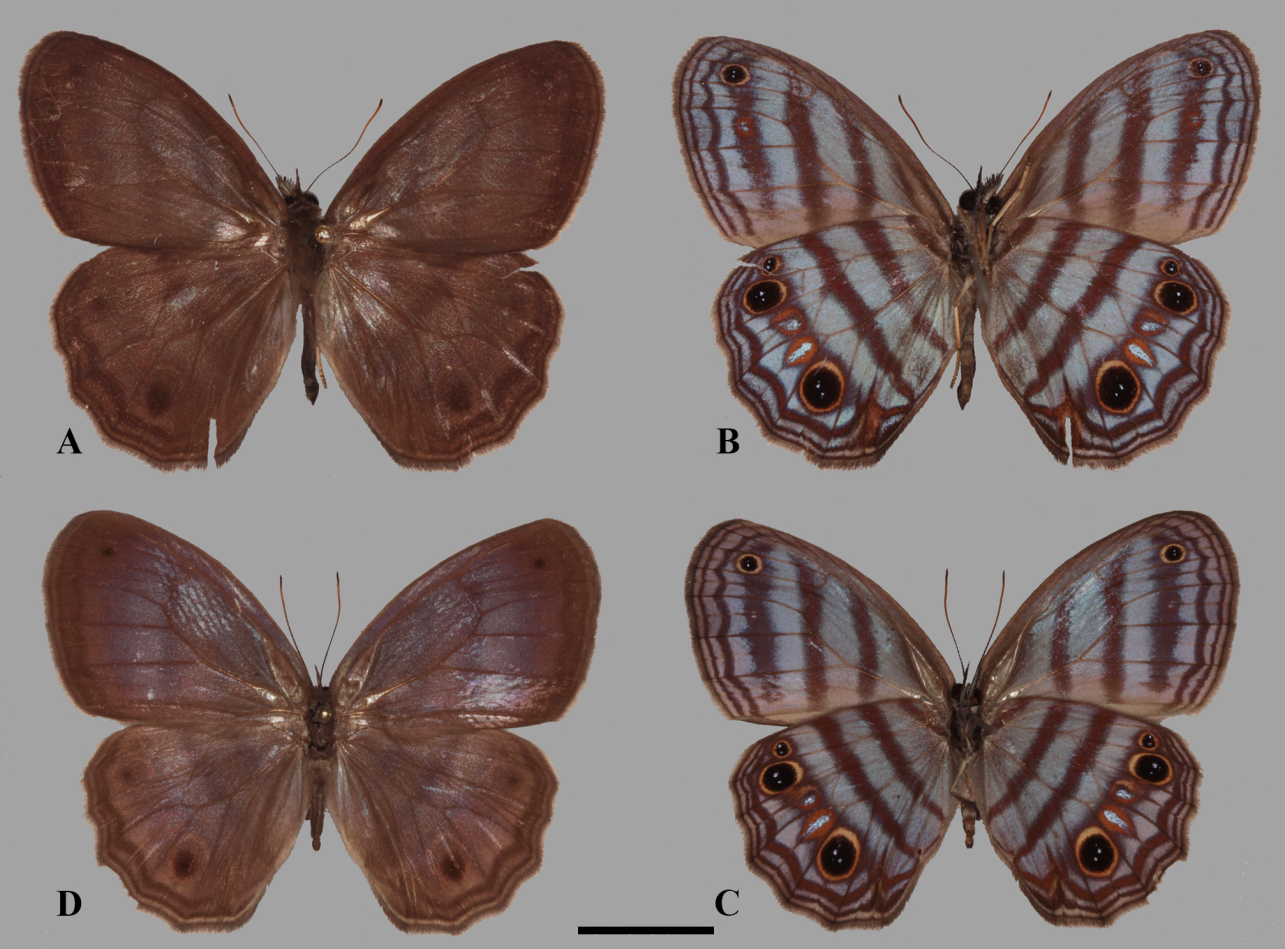
Adult plate. *Scriptor sphenophorus* **n. gen. and n. sp.** male holotype, (A) dorsal, and (B) ventral (MUSM LEP 103507); female paratype, (C) dorsal, and (D) ventral (MUSM LEP 103537). Scale bar = 1 cm.

**Figure 3:**
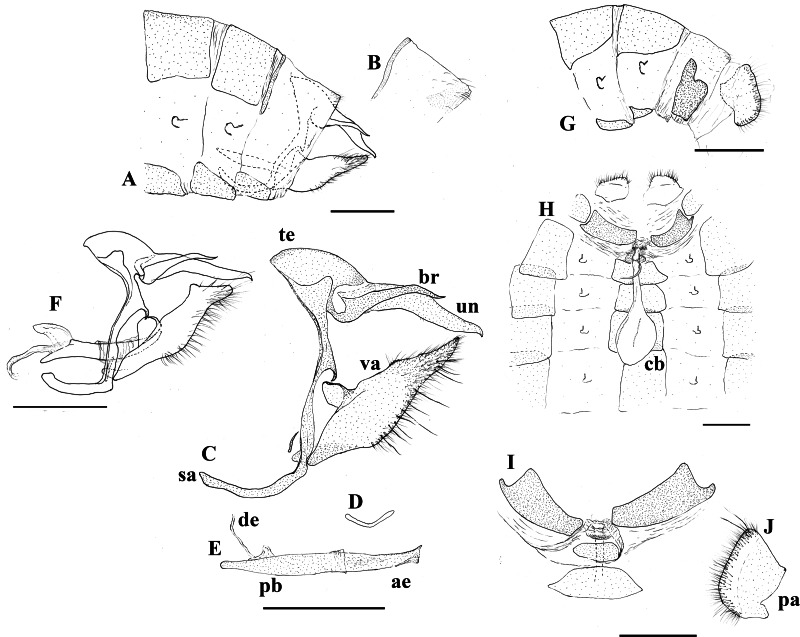
Genitalia plate. *Scriptor sphenophorus* **n. gen. and n. sp.** abdomen and genitalia, MALE based on SN-20-29 (unless indicated otherwise): (A) lateral view of terminal abdominal segments prior to dissection; (B) lateral view of 8th tergite showing broader posterior patch; (C) lateral view of genitalic capsule without phallus; (D) posterior view of juxta; (E) lateral view of phallus; (F) *Magneuptychia ocypete*, lateral view of male genitalic capsule based on SN-20-78 (from Rondônia, Brazil). FEMALE based on SN-20-28 (FLMNH-MGCL-1036473) except for (G), which is based on SN-20-47 (FLMNH-MGCL-1036472): (G) lateral view of terminal abdominal segments prior to dissection; (H) dorsal view of genitalia; (I) Ventral view of 8th abdominal segment; (J) papillae analis. Abbreviations: ae, aedeagus; br, brachium; cb, corpus bursae; de, ductus ejaculatorius; pa, papillae analis; pb, phallobase; sa, saccus; te, tegumen; un, uncus; va, valvae. Scale bar = 1 mm.

**Figure 4:**
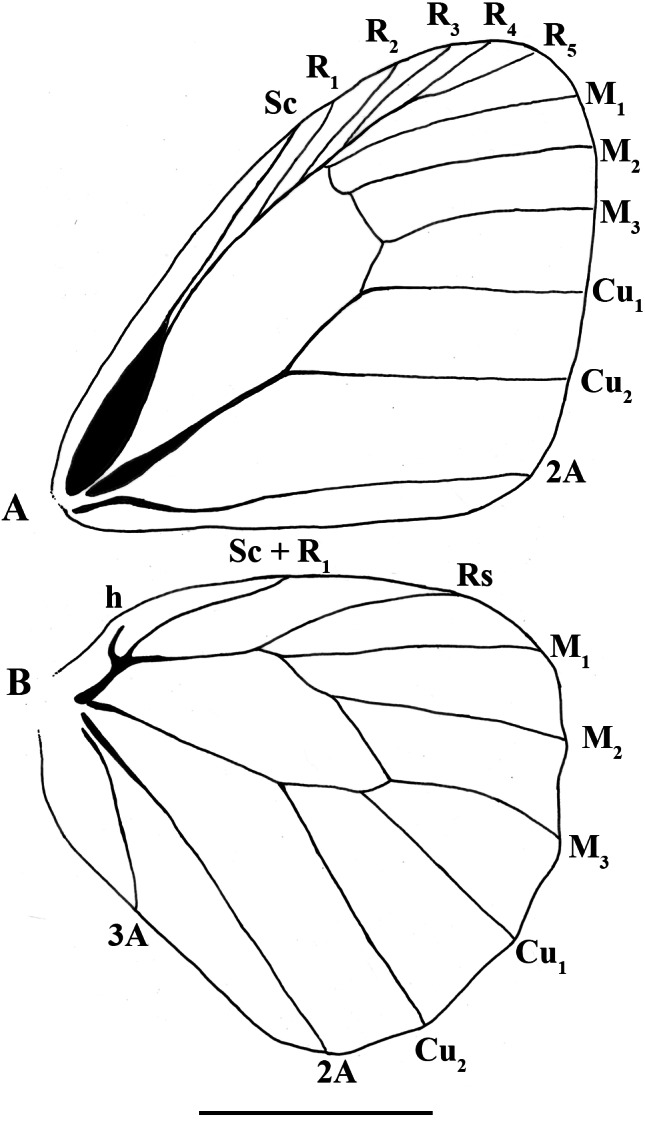
*Scriptor sphenophorus.* n. gen. and n. sp. male wing venation based on FLMNH-MGCL-1036467. (A) Forewing, (B) hindwing. Scale bar = 1 cm.

**Figure 5:**
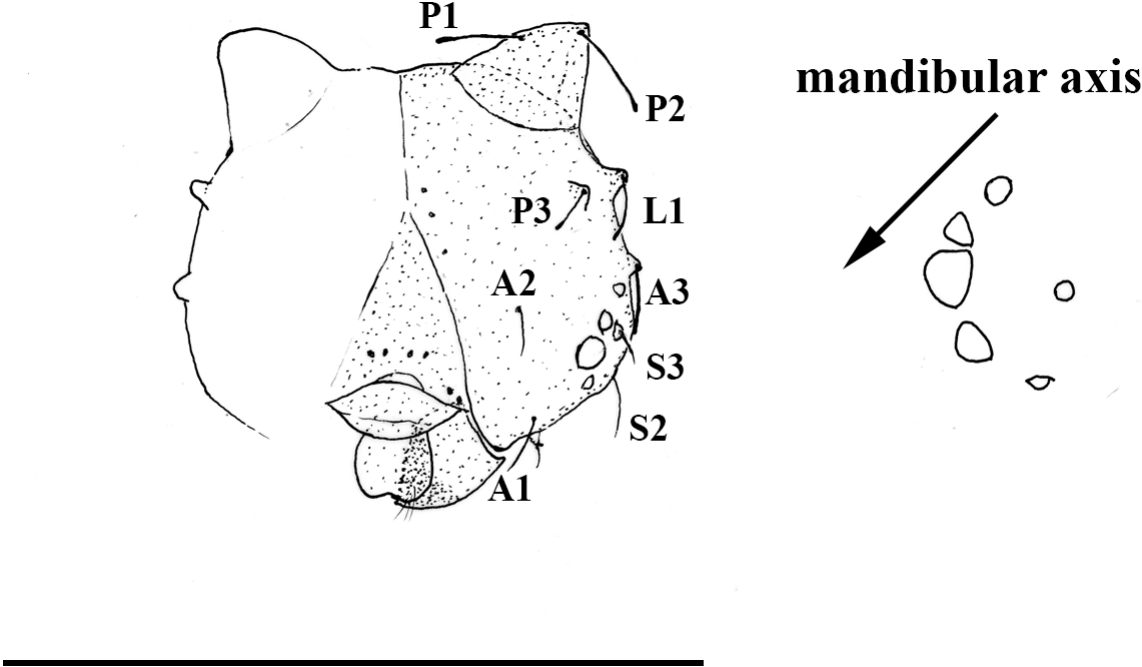
*Scriptor sphenophorus.* n. gen. and n. sp. head capsule and stemmata arrangement based on SN-20-28 (FLMNH-MGCL-1036473). Scale bar = 1 mm.

**Figure 6:**
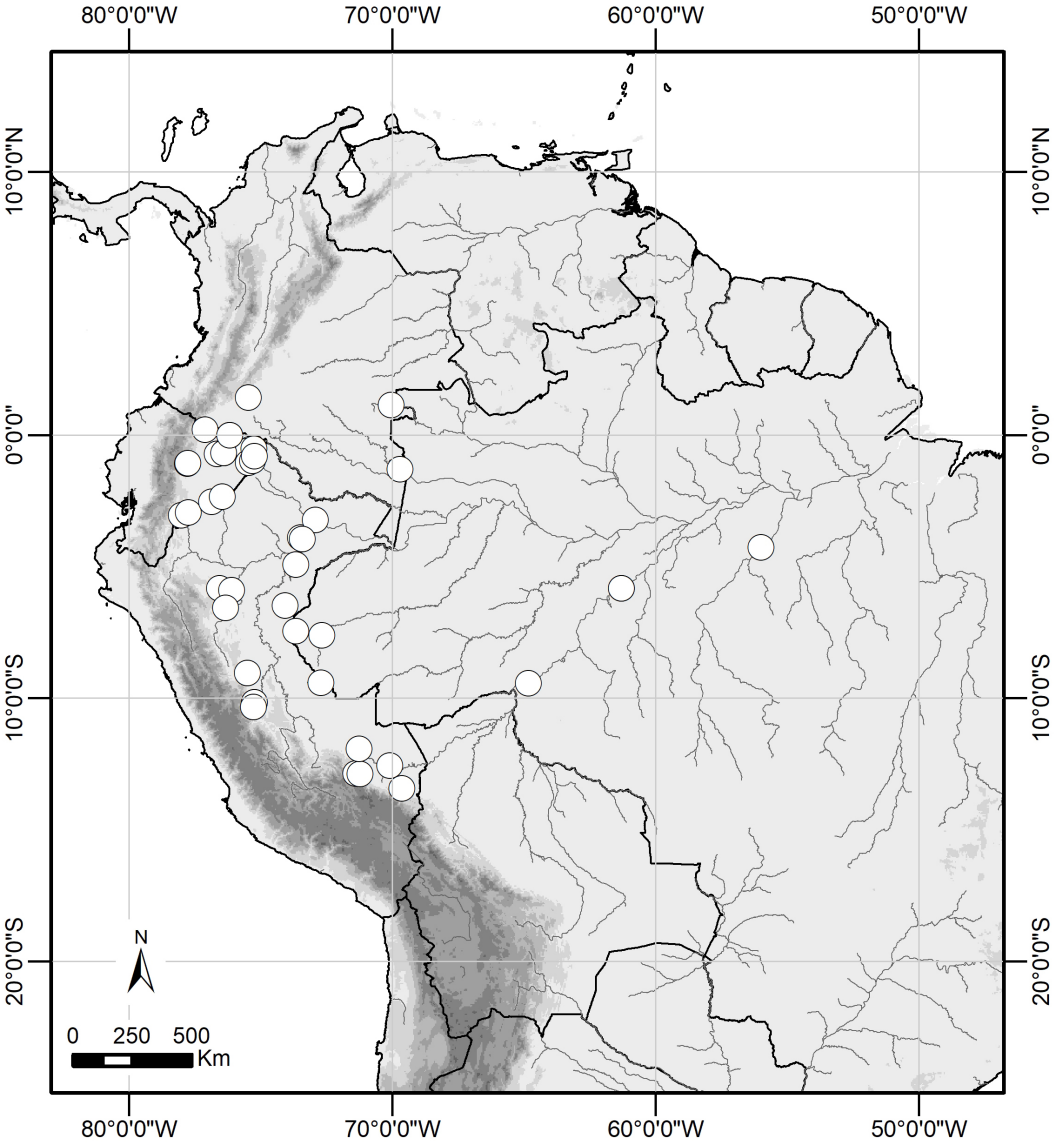
Distribution range of *Scriptor sphenophorus*** n. gen. and n. sp.** in the Amazon basin.

***Scriptor*** Nakahara & Espeland, **new genus**
(Figs. 1–6)
Type species—*Scriptor sphenophorus* Lamas & Nakahara, **n. sp.**, by present designation

**Systematic placement and diagnosis.** *Scriptor* **n. gen.** is a member of the “*Splendeuptychia* clade”, and its sister relationship to the “*ocypete* species group” of *Magneuptychia* is weakly supported (SH-aLRT/UFBoot = 20.4/46; Fig. 1), according to a multi-locus maximum likelihood phylogeny (LnL = − 8762.690; Fig. 1). In all of the ten runs in IQ-TREE, this sister relationship was constantly recovered with low support, and alternative phylogenetic hypotheses are discussed below. The monophyly of *Scriptor sphenophorus* **n. gen. and n. sp.** and the “*ocypete* species group” are both strongly supported by our molecular data (both with UFBoot = 100; Fig. 1). Although recovered as a member of the same “*Splendeuptychia* clade”, the type species of *Magneuptychia*, *Papilio libye* Linnaeus, 1767, does not form a clade with any of the species discussed herein that could reasonably be classified as a single genus (Espeland et al., 2019), excluding the option of describing this species under that generic name. *Scriptor* **n. gen.** is readily distinguished from all other euptychiine genera by the wedge-shaped black marking of the VHW marginal band in cell Cu_2_ which terminates with an inwardly directed, thin black line that crosses the thicker reddish brown submarginal line. However, this wedge-shaped swelling of the marginal band may not be obvious in some specimens (see Variation section below), and it may resemble a similar marking in species in the “*ocypete* species group” of *Magneuptychia*, such as *M. ocypete* (Fabricius, 1776), *M. opima* (Weymer, 1911), *M. louisammour* Benmesbah & Zacca, 2018, as well as *M. sheba* Brévignon & Benmesbah, 2011 (not a member of the ”*ocypete* ” species group). *Scriptor sphenophorus* **n. gen. and n. sp.** is distinguished from these taxa by its elongated ocelli with a single silver spot on the VHW in cells M_2_ and M_3_, whereas these ocelli are more rounded and have double silver spots in otherwise similar species. The VHW submarginal band is reddish throughout in *Scriptor sphenophorus* **n. gen. and n. sp.**, whereas this band in similar species is reddish only at the tornus, except for *M. opima*. Furthermore, the VHW submarginal band is broader in *Scriptor sphenophorus* **n. gen. and n. sp.**, whereas rather narrow (close to the marginal band in width) in similar species, again except for *M. opima*. The male genitalia of *Scriptor sphenophorus* **n. gen. and n. sp.** are easily distinguished from those of species in the “*ocypete* species group” by the lack of a developed “hump” on the dorsal margin of the valva and by only having a slightly serrated region at the dorsal margin distal of the costa. The overall appearance of the valva thus resembles more that of species in the “*fugitiva* species group”, but perhaps has the most pointed posterior tip of the valva compared to *M. fugitiva* Lamas, [1997], *M. kamel* Benmesbah & Zacca, 2018, *C. maripa*, and *C. myncea* (Cramer, 1780). The tapered distal end of the valva seen in *Cissia myncea* is close to *Scriptor sphenophorus* **n. gen. and n. sp.**, but the distal half of the ventral margin is concave in *C. myncea*, whereas rather straight in *Scriptor sphenophorus* **n. gen. and n. sp.** In addition, the apical process of the valva angles in at almost a right angle in dorsal view in *Scriptor sphenophorus* **n. gen. and n. sp.**, whereas in the other species discussed herein it only curves slightly, perhaps with the exception of *M. fugitiva*. The female genitalia of *Scriptor sphenophorus* **n. gen. and n. sp.** are easily distinguished from *M. ocypete* by the lack of sclerotized rectangular lamella antevaginalis extending horizontally and apparently fused with the lateral plate of the eighth abdominal segment (only the area just ventral of ostium bursae is sclerotized in *Scriptor sphenophorus* **n. gen. and n. sp.**).

### Description

**MALE:** Forewing length 21.2–24.4 mm (*n* = 5); Holotype 23.5 mm.

**Head:** Eyes with relatively short sparse light brownish hairs, with white scales at base; frons brown with white scales and semi-iridescent golden brownish elongate scales; first segment of labial palpi short, laterally and dorsally with white scales, ventrally with white long hair-like scales and brownish long hair-like scales, second segment length approximately twice as long as eye depth and covered with white hair-like scales and white scales laterally, and with longitudinal row of black scales present along dorsal surface starting from anterior end of segment, ventrally adorned with black hair-like scales and white hair-like scales longer than segment width, third segment about one-third of second segment in length, porrect, and covered with black scales dorsally and ventrally, with creamy-white scales laterally; antennae approximately two-fifths of forewing length (shorter than discal cell in length), with ca. 39 segments (*n* = 3) including pedicel and scape, distal 12–13 segments composing insignificant club, dark brownish scales apparent more at basal half of antennae with whitish scales at base of each segment.

**Thorax:** Brown, dorsally and laterally covered sparsely with greyish scales and additionally with long, dense light brownish hair-like scales; ventrally scattered with greyish scales and with long whitish hair-like scales; foreleg greyish, with long creamy hair-like scales, whitish tarsus, and tibia almost equal in length, femur slightly longer; pterothoracic leg femur with whitish grey scales, basal two-thirds adorned with long greyish hair-like scales ventrally, tibia and tarsus ocher, dorsally appearing darker, dorsal surface of tarsal segments appearing darker, tibia with two longitudinal rows of three long spines ventrally, as well as few spines present laterally, tarsus and tibia adorned with spines ventrally, tibial spurs present at distal end of tibia (two spurs equal in length), tarsus with three longitudinal rows of spines ventrally until distal end of first tarsomer, rows of spines increasing to four after this point.

**Wing venation** (Fig. 4)**:** Basal half of forewing subcostal vein swollen; base of cubitus swollen; forewing recurrent vein absent; disco-cellular vein m_1_-m_2_ curved inwards; hindwing humeral vein developed; origin of M_2_ slightly closer to M_1_ than to M_3_.

**Wing shape:** Forewing overall appearing subtriangular, apex rounded, costal margin convex, outer margin almost straight except for curved around M_1_ as part of rounded apex, inner margin straight, but rounded towards thorax near base; hindwing overall appearing rounded and somewhat elongate, costal margin convex, outer margin slightly undulating, tornus rounded, inner margin convex but curving inwards near base.

**Dorsal forewing:** Ground colour brownish, distally darker, subtly translucent and thus somewhat revealing ventral bands, dark-brownish submarginal band extending from apex towards tornus, dark-brownish marginal band extending from apex towards tornus.

**Dorsal hindwing:** Ground colour and general wing pattern similar to forewing, submarginal and marginal bands both undulating, streak derived from marginal line in cell Cu_2_ visible in some specimens, darker spots in cell M_1_ and Cu_1_ mirroring ventral ocelli.

**Ventral forewing:** Ground colour purplish grey; band absent along swollen subcostal vein; reddish-brown discal band extends from radial vein (just distal of origin of R_1_), crossing discal cell in a slightly inward diagonal direction and somewhat outwards below cubital vein, overall appearing curved basally, fading in cell Cu_2_; concolourous postdiscal band extending from radial vein (near origin of R_3_) towards 2A, wider and appearing straight compared to previous band, passing origin of Cu_1_ and terminating at 2A; umbra appearing as dark irregular band extending from around branching of R_4_ and R_5_, similar width or broader compared to previous band, terminating in cell Cu_2_; reddish-brown well-defined sinuate submarginal band extending from apex towards tornus, broadening in cells M_3_, Cu_1_, and to some extent Cu_2_, width about as half as discal band, narrowing and terminating shortly after passing 2A; smooth concolourous marginal band, narrower than previous band, extending from apex towards tornus; fringe brownish; ocellus in cell M_1_, black with two white pupils in centre, ringed in yellow (but see “variation” section below).

**Ventral hindwing:** Ground colour similar to forewing; short reddish-brown band near wing base; concolourous discal band similar in width as VFW discal band, extending from costal margin towards inner margin, somewhat narrowing posteriorly; concolourous postdiscal band almost parallel to discal band, extending from costal margin to inner margin passing origin of Cu_2_, evenly broad as previous band, concolourous scaling extending to origin of M_2_ and extending beyond in some specimens (but see “variation” section below), anterior end occasionally extending distally along costa and posterior end bent basally along inner margin; umbra similar to that of VFW in appearance, surrounding submarginal ocelli; reddish-brown submarginal band similar to that of VFW in appearance, broader, appearing slightly broadening towards tornus, strongly bent inwards in cell Cu_2_, anterior end and posterior end occasionally fused with postdiscal band in cell Rs and 2A, respectively (posterior end more so than anterior end); marginal band, darker, undulate, similar to that of VFW in colour and width, but broadening in cell Cu_2_ and2A, streak derived from this broadened region, penetrating submarginal band and reaching postdiscal band in some specimens resulting in wedge-shaped swelling, in addition to small dark streak visible in cell Cu_2_ just basal of submarginal band in some specimens (see “variation” section below); fringe brownish; submarginal ocelli in cells Rs, M_1_, M_2_, M_3_ and Cu_1_, those in cells Rs, M_1_, and Cu_1_ similar in appearance to forewing ocellus in cell M_1_, ocellus in Rs smaller than those in M_1_ and Cu_1_ (these two ocelli touching veins defining cells), ocelli in cells M_2_ and M_3_ appearing somewhat oval, with single silver spot surrounded by orangish broad ‘ring’, tapering distally.

**Abdomen:** Eighth tergite reduced, appearing only along basal margin of dorsal surface of eighth abdominal segment, broad patch on distal side apparently split into two patches (Figs. 3A and 3B); eighth sternite present as a single plate.

**Genitalia** (Figs. 3C–3E): Tegumen appears somewhat semi-circular in lateral view with rather moderately convex dorsal margin, slightly elongated in lateral view, ventral margin appearing roughly straight in lateral view; uncus about as twice as long as tegumen in lateral view, slightly bent in middle and tapered towards posterior end in lateral view, posterior end slightly hooked downwards, appearing spatulate in dorsal view with angular posterior tip, short hair-like setae visible ventrally towards base; brachia appearing about three-fourths of uncus in length viewing laterally, appearing almost parallel to uncus in dorsal view, apical point appear higher than uncus in lateral view, apex slightly hooked upwards; combination of ventral arms from tegumen and dorsal arms from saccus slightly curving in middle; appendices angulares present, curving downwards towards costa; saccus narrow, 1.5x longer than tegumen in length viewed laterally; juxta present, shallow ‘V’-shaped plate in posterior view; distal half of valve setose with some noticeably thickened setae present, roughly parallelogram-shaped in lateral view, basally and distally slightly elongated, apical process in particular protruding and slightly curving towards uncus, costa appearing somewhat pentagonal, projecting towards appendices angulares from dorsal margin of valva, ventral margin of costa fully attached to valva, serrated region present distal of costa on dorsal margin; phallus slightly longer than uncus in lateral view, appearing straight, phallobase occupying approximately half of phallus, ductus ejaculatorius visible, very slightly sclerotized region of vesica visible through aedeagus, vesica visible at postero-ventral opening of aedeagus.

**FEMALE:** Forewing length 20.5–21.5 mm (*n* = 3)

**Similar to male except as follows:** Foretarsus divided into five distinctive (i.e., not fused) tarsomers; forewing generally appearing more rounded and broader; ground colour of dorsal wing surface paler, thus bands and dark spots appear generally more defined; purple semi-metalic sheen visible dorsally; small ocellus present at posterior end of postdiscal band in a few specimens (see “variation” section below). **Female abdomen and genitalia** (Figs. 3F–3I): Eighth tergite fully developed but apparently weakly sclerotized; papillae analis with rounded posterior apophysis; lamella antevaginalis sclerotized, but sclerotized region limited to just ventral area of ostium bursae (i.e., where ductus bursae meets ventral surface of 8th abdominal segment); sclerotized plate of 8th abdominal segment present laterally, not fused with lamella antevaginalis; weakly sclerotized region apparently present ventrally in intersegmental membrane of seventh and eighth abdominal segments, this intersegmental membrane only partially pleated and expandable (compared to fully expandable membrane of many other euptychiines); ductus bursae membranous, ductus seminalis exits dorsally at one-fifth distance from ostium bursae to corpus bursae, ductus bursae slightly inflated where origin of ductus seminalis exits; corpus bursae roughly oval in dorsal view, extending to juncture of fourth and fifth abdominal segment, with two rather narrowly appearing signa present, parallel to each other, located posteriorly.

**Variation.** As indicated above, this taxon exhibits some recognizable intra-specific variation which includes: (1) extra ocellus or ocelli can be present in adjacent cell(s) below VFW ocellus in cell M_1_ (e.g., MUSM-LEP 103506; 103534); (2) width of ventral bands can appear broad in some specimens (e.g., MUSM-LEP 103511; 103513; 103514), whereas narrow in some individuals (e.g., MUSM LEP 103521; 103546; 103552); (3) scales extending along discal cell vein towards origin of M_2_ from VHW postdiscal band in some individuals (e.g., MUSM-LEP 103514; 103519); (4) VHW wedge-shaped swelling at tornus reaching postdiscal band in some specimens (e.g., MUSM-LEP 103513; 103504), whereas in some individuals this swelling does not extend further beyond submarginal band (e.g., MUSM-LEP 103519; 103544); (5) Although apparently only restricted to few female specimens (e.g., MUSM-LEP 103537; 103543), a small ocellus may appear at posterior end of VHW postdiscal band.

**Immature stages.** A first instar larva was found during the dissection of one female specimen (SN-20-28, from Amazonas, Brazil) and the head capsule is illustrated in Fig. 5. Notable features include: primary setae appearing rather hair-like (unlike the spatulate setae of species in the so-called “*Taygetis* clade”) with slightly dilated apex; developed scoli present with two setae; six chalazae present; six stemmata present with 3rd stemma being the largest.

**Etymology.** The generic name is a Latin masculine noun in the nominative singular meaning “writer” or “scribe”, in reference to the VHW wedge-shaped streak and associated black line derived from the marginal line in cell Cu_2_ being somewhat reminiscent of a pencil marking.

**Table:** 

***Scriptor sphenophorus*** Lamas & Nakahara, **new species**
*Magneuptychia* sp. n. 1 - Robbins et al. (1996: 231); Lamas, Robbins & Harvey (1997: 65)
*Magneuptychia* n. sp. Lamas, MS - Lamas (2004: 220)

**Systematic placement and diagnosis.** Consult the corresponding section above.

**Description.** Consult the description provided for the genus above.

**Types.** HOLOTYPE, male with following labels written verbatim and separated by forward slashes: // PERU, Loreto, Arcadia 0°59.37′S, 75°18.55′W 150 m, 10 Nov 1993 leg. G. Lamas // Photographed By K. Willmott June 2015// MUSM-LEP 103507// (MUSM)

**PARATYPES (60 M, 51 F)**: **Brazil**: *Acre*: Mancio Lima, Parque Nacional Serra do Divisor, Porção Norte, [7°26′50″S,73°39′52″W], 200-400 m, (Dolibaina, D., Moura, D.), 10-21 Sep 2011, 1 ♂, (DZUP); Reserva Extrativista Alto Juruá, Marechal Thaumaturgo, Foz do Rio Breu, [9°24′35″S,72°42′58″W], 200-300 m, (Brown, K. S., Freitas, A. V. L.), Oct 1997, 1 ♂, (ZUEC); Rio Juruá, Cruzeiro do Sul, [7°37′S,72°40′W], 200 m, 17 Feb 1976, 1 ♀, (DZUP); *Amazonas*: Cucuí, (d’Almeida, R. F.), Jul 1949, 1 ♀, (DZUP); Manicoré, [5°49′S,61°17′W], 16 Aug 1976, 1 ♀ [FLMNH-MGCL-1036473; dissection, SN-20-28], (FLMNH), (Boy, H. C.), (ZSM), Jun, (ZSM), (Callaghan, C.), 16 Aug 1976, 1 ♂ [FLMNH-MGCL-1036467; dissection, SN-20-29], (FLMNH); *Pará*: [Rio] Tapajós, [4°16′8″S,55°59′10″W], 25 m, 1 Dec 2012, 1 ♂, (ZUEC), 20 Sep 2013, 1 ♂, (ZUEC), 6 Nov 2013, 1 ♂, (ZUEC); *Rondônia*: Porto Velho, Lago Jirau, [Igarapé] Caiçara, [9°26′19″S,64°50′W], 250 m, 19 Jun 2012, 1 ♂, (ZUEC). **Colombia**: *Amazonas*: Río Caquetá, La Pedrera, [1°18′S,69°42′W], 170 m, 23 May 1992, 1 ♀ [FLMNH-MGCL-1036470; dissection, SN-14-182], (FLMNH), May 1992, 1 ♂ [FLMNH-MGCL-1036468; dissection, SN-14-183], 1 ♀ [FLMNH-MGCL-1036472; dissection SN-20-47], (FLMNH); *Caquetá*: Montañita, [1°25′N,75°28′W], 366 m, (Nicolay, S. S.), 26 Jan 1971, 1 ♂ [FLMNH-MGCL-1036465], (FLMNH); *Vaupés*: N[orth?]. Mitú, [1°8′N,70°3′W], 200 m, (Simon, M.), 5 Aug 1983, 1 ♂ [FLMNH-MGCL-1036466], (FLMNH). **Ecuador**: *Morona-Santiago*: hill N of Santiago, [3°2′51″S,78°0′23″W], 350 m, (Hall, J. P. W.), 24-25 Feb 2017, 1 ♀ [FLMNH-MGCL-281451], (FLMNH); km 47.6 Santiago-Puerto Morona rd., [2°56′12″S,77°44′48″W], 245 m, (Busby, R. C.), 7 Jan 2015, 1 ♂ [FLMNH-MGCL-195446], (FLMNH); *Napo*: Río Sinde, km 12 Tena-Puyo rd., Finca San Carlo, [1°5′18″S,77°47′24″W], 600 m, (Willmott, K. R., Hall, J. P. W.), 14 Apr 1995, (KWJH); Sinde, [1°4′30″S,77°46′18″W], 600 m, (Perceval, M. J.), 24 Oct 1997, 1 ♂, (MIPE); *Orellana*: Laguna Zancudococha, military trail, [0°35′16″S,75°28′16″W], 220 m, (Aldaz, R.), 9-13 Jul 2017, 1 ♂ [FLMNH-MGCL-288755], 1 ♂ [FLMNH-MGCL-288757], (FLMNH); lower Río Yasuní, ’Pichincha’ trail, [1°3′11″S,75°27′53″W], 190 m, (Willmott, K. R., J. C. R., J. I. R.), 30 Jun 2017, 1 ♀ [FLMNH-MGCL-288756], (FLMNH); Parque Nacional Yasuní, 10 km E Guardianía Pindo, [0°43′6″S,76°39′8″W], 330 m, (Hall, J. P. W., Willmott, K. R., J. C. R., J. I. R), 21,22 Jul 2016, 1 ♂ [FLMNH-MGCL-209874], 1 ♀ [FLMNH-MGCL-209869], (FLMNH); Río Tiputini, Estación Científica Yasuní, parcela 50 Ha, [0°40′55″S,76°24′1″W], 250-270 m, (Willmott, K. R., J. I. R., J. C. R., Páez, E.), 5 Jul 2014, 1 ♀ [FLMNH-MGCL-195182], (FLMNH);Río Tiputini, vía Auca, Estación Científica Yasuní, [0°40′27″S,76°23′49″W], 220-250 m, (Robinson Willmott, J. I., J. C.), 5 Jul 2014, 1 ♂ [FLMNH-MGCL-195186], 1 ♀ [FLMNH-MGCL-195183], 1 ♀ [FLMNH-MGCL-195184], 1 ♀ [FLMNH-MGCL-195185], (FLMNH), (Willmott, K. R., J. I. R., J. C. R.), 6 Jul 2014, 1 ♂ [FLMNH-MGCL-195187], (FLMNH); Estación Científica Yasuní, [0°40′27″S,76°23′49″W], 250 m, (Grados, J.), 3 Dec 2004, 1 ♂ [MUSM-LEP-103504], (MUSM); Río Tiputini, víaAuca, Estación Científica Yasuní, [0°40′27″S,76°23′49″W], 300 m, (Hall, J. P. W.), 23-28 Feb 2018 [FLMNH-MGCL-297421] [FLMNH-MGCL-297422], (FLMNH); *Pastaza*: Río Capahuari, Kapawi Lodge, [2°32′30″S,76°51′32″W], 250 m, (Willmott, K. R., Hall, J. P. W.), 21,22,27 Jul 2009, 1 ♀ [FLMNH-MGCL-145666], (FLMNH); Yutsuntsa, [2°21′4″S,76°27′14″W], 250 m, (Nakahara, S.), 12 Jul 2014, 1 ♀, (FLMNH); *Sucumbíos*: Cuyabeno Lodge, across lagoon, [0°0′18″S,76°10′23″W], 224 m, (Turner, J. D.), 5 Dec 2010, 1 ♀ [FLMNH-MGCL-150973], (FLMNH); Cuyabeno, Reserva de Producción Faunística, [0°0′4″S,76°10′50″W], 230 m, (Kareofelas, G., Witham, C. W.), 20 Nov-12 Dec 1993, 1 ♂, (BME); Lumbaquí-Lago Agrio rd., 15 km N Sevilla, [0°12′26″N,77°6′59″W], 380 m, (Hall, J. P. W., Willmott, K. R., J. C. R., J. I. R), 17,19 Jul 2016, 1 ♀ [FLMNH-MGCL-209871], (FLMNH); *Not located*: ‘Ecuador’, (Nakahara, S.), 2014, (FLMNH). **Peru**: *Loreto*: Aguas Negras, [0°31′24″S,75°15′24″W], 150 m, (Lamas, G.), 1 Mar 1994, 1 ♂ [MUSM-LEP-103511], (MUSM), 4 Mar 1994, 1 ♂ [MUSM-LEP-103508], 1 ♀ [MUSM-LEP-103532], (MUSM), (Robbins, R. K.), 1 Mar 1994, 1 ♂ [MUSM-LEP-103509], (MUSM), 6 Mar 1994, 1 ♂ [MUSM-LEP-103510], (MUSM); Arcadia, [0°59′22″S,75°18′33″W], 150 m, (Lamas, G.), 6 Nov 1993, 1 ♀ [MUSM-LEP-103559], (MUSM), (Robbins, R. K.), 4 Nov 1993, 1 ♀ [MUSM-LEP-103533], (MUSM); Balsapuerto, [5°50′S,76°34′W], 220 m, (Klug, G. G.), Feb 1939, 1 ♂ [MUSM-LEP-103505], (MUSM); Castaña, [0°48′S,75°14′W], 150 m, (Lamas, G.), 18 Oct 1993, 1 ♀ [MUSM-LEP-103561], (MUSM), 19 Oct 1993, 1 ♀ [MUSM-LEP-103560], (MUSM), 24 Oct 1993, 1 ♀ [MUSM-LEP-103536], (MUSM), (Robbins, R. K.), 20 Oct 1993, 1 ♀ [MUSM-LEP-103535], (MUSM); Explornapo-ACEER, [3°14′S,72°55′W], 140 m, (Caldas, A.), 13 Sep 1995, 1 ♀ [MUSM-LEP-103526], 1 ♀ [MUSM-LEP-103527], (MUSM), 16 Sep 1995, 1 ♀ [MUSM-LEP-103555], (MUSM), 18 Sep 1995, 1 ♀ [MUSM-LEP-103558], (MUSM), (Grados, J.), 11 Sep 1995, 1 ♀ [MUSM-LEP-103542], (MUSM), 14 Sep 1995, 1 ♀ [MUSM-LEP-103538], 1 ♀ [MUSM-LEP-103564], (MUSM), 19 Sep 1995, 1 ♀ [MUSM-LEP-103541], (MUSM), 4 Sep 1995, 1 ♀ [MUSM-LEP-103525], (MUSM), 6 Sep 1995, 1 ♀ [MUSM-LEP-103543], (MUSM), 7 Sep 1995, 1 ♂ [MUSM-LEP-103512], 1 ♀ [MUSM-LEP-103565], (MUSM), 9 Sep 1995, 1 ♀ [MUSM-LEP-103556], (MUSM), (Harvey, D. J.), 10 Sep 1995, 1 ♀ [MUSM-LEP-103566], (MUSM), 16 Sep 1995, 1 ♀ [MUSM-LEP-103562], (MUSM), 4 Sep 1995, 1 ♂ [MUSM-LEP-103513], (MUSM), 9 Sep 1995, 1 ♀ [MUSM-LEP-103539], 1 ♀ [MUSM-LEP-103540], (MUSM), (Robbins, R. K.), 5 Sep 1995, 1 ♀ [MUSM-LEP-103557], 1 ♀ [MUSM-LEP-103563], (MUSM); Jenaro Herrera, [4°55′S,73°40′W], 125 m, (Lamas, G.), 13 Aug 2013, 1 ♀ [MUSM-LEP-103567], (MUSM); Río Nanay, Mishana, Estación Biológica Callicebus, [3°54′S,73°29′W], 150 m, (Lamas, G.), 10 Jan 1980, 1 ♀ [MUSM-LEP-103534], (MUSM); Yurimaguas, [5°54′S,76°6′W], 120 m, (Michael), 1898, 1 ♂, (MNHU); Zona Reservada Allpahuayo-Mishana, [3°57′30″S,73°25′30″W], 170 m, (Campos, L.), 19 Feb 2002, 1 ♀ [MUSM-LEP-103524], (MUSM), (Ramírez, J. J.), 20 Feb 2002, 1 ♂ [MUSM-LEP-103522], 1 ♀ [MUSM-LEP-103537], (MUSM), 27 Nov 2001, 1 ♂ [MUSM-LEP-103514], (MUSM); *Madre de Dios*: Erika, [12°53′S,71°14′W], 550-650 m, (Lamas, G.), 4-5 Sep 1989, 1 ♀ [MUSM-LEP-103521], (MUSM); Los Amigos Biological Station, [12°34′2″S,70°5′56″W], 270 m, (Gallice, G.), 18 Nov 2012, 1 ♂ [MUSM-LEP-103520], (MUSM), (Peña, C.), 8 Jul 2003, 1 ♂ [MUSM-LEP-103515], (MUSM); Parque Manu, Pakitza, [11°55′48″S,71°15′18″W], 340 m, (Clarke, N. L.), 3 May 1991, 1 ♀ [MUSM-LEP-103545], (MUSM), (Harvey, D. J.), 22 Apr 1991, 1 ♂ [MUSM-LEP-103517], (MUSM), (Mielke, O. H. H.), 17 Oct 1991, 1 ♀ [MUSM-LEP-103544], (MUSM), 19 Oct 1991, 1 ♀ [MUSM-LEP-103550], (MUSM), 9 Oct 1991, 1 ♂ [MUSM-LEP-103518], (MUSM); Parque Manu, Pakitza, [11°55′48″S,71°15′18″W], 400 m, (Lamas, G.), 11 Oct 1990, 1 ♀ [MUSM-LEP-103549], (MUSM), 21 Oct 1990, 1 ♀ [MUSM-LEP-103548], (MUSM), 3 Oct 1990, 1 ♀ [MUSM-LEP-103547], (MUSM), (MacDonald, J.), 27 Oct 1990, 1 ♂ [MUSM-LEP-103516], (MUSM), (Robbins, R. K.), 6 Oct 1990, 1 ♀ [MUSM-LEP-103528], (MUSM), (Rowe, W.), 10 Nov 1990, 1 ♀ [MUSM-LEP-103546], 1 ♀ [MUSM-LEP-103551], (MUSM), 30 Oct 1990, 1 ♀ [MUSM-LEP-103552], (MUSM), 4 Nov 1990, 1 ♀ [MUSM-LEP-103553], (MUSM); Río Madre de Dios, Albergue Amazonia, [12°52′S,71°23′W], 500 m, (Gibson, L.), 28 Oct 2010, 1 ♂ [MUSM-LEP-103519], (MUSM); Albergue Pantiacolla, [12°39′71°14′W], 400-450 m (Lamas, G.), 26 Oct 2016, 1 ♂ (MUSM); 28 Oct 2016, 3 ♀ (MUSM); 29 Oct 2016, 1 ♀ (MUSM); 400 m (Kinyon, S.), 2 Nov 2018, 1 ♀ (MUSM); *Pasco*: Chuchurras, [10°9′S,75°14′W], 300 m, 1 ♀ [FLMNH-MGCL-1036471], (FLMNH), (Martin, P.), 1 ♀, (CMNH); Parque Nacional Yanachaga-Chemillén, Paujil, [10°20′S,75°16′W], 500 m, (Icochea, J.), 21 Oct 1993, 1 ♂ [MUSM-LEP-103506], (MUSM); *Puno*: Tambopata-Candamo, Río Távara, [13°26′S,69°38′W], 300-1050 m, (Grados, J.), 12 Aug 1995, 1 ♀ [MUSM-LEP-103529], (MUSM); Tambopata-Candamo, Río Távara, [13°26′S,69°38′W], 450–1050 m, (Cambridge University Amazon Expedition), 1995, 1 ♀ [MUSM-LEP-103530], (MUSM), (Grados, J.), 31 Jul 1995, 1 ♀ [MUSM-LEP-103531], (MUSM); *San Martín*: Tarapoto - Yurimaguas, km 20, [6°34′S,76°20′W], 950 m, (Lamas, G.), 21 Nov 2007, 1 ♀ [MUSM-LEP-103554], (MUSM); *Ucayali*: Aguaytía, [9°3′S,75°30′W], 400 m, (Foerster, J.), 23 Aug 1961, [sex cannot be determined based on images] (ZSM);32 km E Monte Alegre, Río Tapiche, [6°28′31″S,74°4′32″W], 139 m, (García, A.), 14-15 Oct 2008, 1 ♀ [MUSM-LEP-103523], (MUSM). **Country unknown**: *Not located*: no data, 1 ♂ [FLMNH-MGCL-1036469; dissection, SN-14-185], (FLMNH).

**Other records**: **Ecuador**: *Orellana*: Río Tiputini, Estación Científica Yasuní, parcela 50 Ha, [0°40′55″S,76°24′1″W], 250-300 m, (Mena, S.), 2017, 2 ♂, 8 ♀, (PUCE) (Checa, M. F. (Oct 2017, pers. comm. by email with photo to KRW)).

**Etymology.** This specific epithet is derived from “*sphenophorus*”, a Greek word meaning “wedge-bearing”, alluding to the wedge-shaped swelling of the VHW tornus. The species-group name is regarded as a Latinized masculine noun in the nominative singular standing in apposition to the generic name.

**Distribution and natural history.** *Scriptor sphenophorus* **n. gen. and n. sp.** is likely widely distributed across the Amazon basin, although we have examined specimens only from Colombia, Ecuador, Peru and Brazil (Fig. 6). The species occurs in primary to slightly disturbed lowland rainforest and has been recorded up to 950 m, although it is more commonly found below 500 m. In Ecuador and Peru, both sexes were found in the understory of slightly hilly *terra firme* forest, flying and resting less than 1 m above the ground, with males observed in the mid- to late morning and females in the early afternoon.

## Discussion

The generic status of *Scriptor* **n. gen.** is supported by the maximum likelihood phylogeny generated for this study, in addition to a comprehensive, unpublished molecular phylogeny of Euptychiina, which includes over 2,000 individuals representing over 420 euptychiine species (Espeland et al. in prep.). This latter study recovered *Scriptor* **n. gen.** as sister to *Colombeia* Viloria, Andrade & Le Crom, 2019, although again with low support. We regard the weak support for *Scriptor* **n. gen.** as sister to either the “*Magneuptychia ocypete* species group” or *Colombeia*, and the lack of other strong morphological support for its association with either of these groups of species, as sufficient reason for description of a monotypic genus, to provide a stable classification. Clearly, this taxonomic decision is subjective, and an alternative might be to recognize a much larger genus to accommodate all of these species. Nevertheless, such a broad generic classification would be inconsistent with current approaches to building an informative generic classification for the Euptychiina, where monophyly, phenotype and ecological information are incorporated in deciding on the most appropriate taxonomy (e.g., Nakahara et al., 2019b). For example, we envisage *Colombeia* as including several species whose habitats range from lowland (west of the Andes) to cloud forest (east of the Andes), in addition to somewhat varying overall appearance. *Colombeia* was established as a monotypic genus by Andrade et al. (2019) to harbor *Euptychia mycalesis* Röber, 1927, although this approach was based solely on “interpretation” of their morphology and the hypothesis was not explicitly tested with a phylogenetic analysis. Our molecular data suggest that *Colombeia* should be expanded to include several additional taxa, and these taxonomic changes will be the focus of a forthcoming paper.

There will likely be some debate about the justification for describing yet another monotypic euptychiine genus. Since the beginning of a broad collaborative project to revise Euptychiina systematics (https://www.floridamuseum.ufl.edu/museum-voices/euptychiina), ten monotypic euptychiine genera have been erected (Freitas et al., 2013; Freitas et al., 2016; Freitas et al., 2019; Costa et al., 2016; Nakahara et al., 2015; Nakahara et al., 2016; Nakahara et al., 2018a; Nakahara et al., 2019a; Andrade et al., 2019), although some of these studies did not use phylogenetic analyses to support the proposed taxonomy, as mentioned above. *Scriptor* **n. gen.** represents another monotypic genus for the subtribe, and along with several other monotypic genera awaiting to be described, approximately 30% of euptychiine genera will be monotypic. Martins, Duarte & Robbins (2019: 126), for example, stated that “the information content of monotypic genera is redundant (Farris, 1979)”^1^ 1Note that the reference for Farris, 1979 was not provided in Martins, Duarte & Robbins (2019).and “…monotypic genera are not testable hypotheses”. The first of these claims seems to be a misinterpretation; Farris (1976: 275) argued that monotypic taxa were not “well-justified”, but nevertheless stated that monotypic genera were clearly needed for a species that would otherwise be assigned to no genus, because the rules of binominal nomenclature require every species to be placed in a genus. Such cases arise if a species is found to be sister to an existing genus (and inclusion of the species in that genus reduces the heuristic value of the generic name) or to a clade containing multiple genera, as is the case with *Scriptor* **n. gen**. Wiley (1979) also argued against the unnecessary creation of monotypic higher taxa, but similarly recognized that monotypic genera were an exception, for the same reasons as Farris (1976). We further disagree that a monotypic genus is an untestable hypothesis; on the contrary, a monotypic genus reflects the hypothesis that the constituent species is not nested within any other recognized genus, which can obviously be tested with phylogenetic methods. Clearly, as stated by Farris (1976), one should not establish monobasic taxa because of their “(morphological) distinctiveness” alone, since virtually all practicing taxonomists now recognize and agree that taxa should also be monophyletic. The reluctance of some taxonomists (e.g., Martins, Duarte & Robbins, 2019) to accept monotypic genera is likely partly the result of the inadvisable practice of naming genera without a phylogenetic analysis (e.g., Andrade et al., 2019; González et al., 2019; Viloria & Luis-Martínez, 2019). The fact that genera described in such a way often later prove to be invalid is no argument against the concept of the monotypic genus *per se*. As shown in the present study and some of the references cited above (e.g., Nakahara et al., 2015; Nakahara et al., 2016), phylogenetic analyses reveal a number of lineages in Euptychiina that warrant monotypic generic status in order to maintain the monophyly of other genera and satisfy the rules of binominal nomenclature, although obviously any higher classification is subjective.

## Conclusion

A new monotypic satyrine butterfly genus to accommodate a new species has been erected based on a multi-locus maximum likelihood phylogeny.

## Additional Information and Declarations

### Field Study Permissions

The following information was supplied relating to field study approvals (i.e., approving body and any reference numbers): INABIO and Ecuadorian Ministerio del Ambiente arranged necessary permits for research in Ecuador, most recently under the project ‘Diversity and Biology of Lepidoptera in Ecuador’ (No. 006-19 IC-FLO-FAU-DNB/MA).

### DNA Deposition

The following information was supplied regarding the deposition of DNA sequences: Data is available at GenBank: MT787241 to MT787285.

### Data Availability

The following information was supplied regarding data availability: Specimens are housed at Bohart Museum of Entomology, University of California Davis, Davis, USA; Carnegie Museum of Natural History, Pittsburgh, USA; Entomological Collection Padre Jesus Santiago Moure, Departamento de Zoologia, Universidade Federal do Paraná, Curitiba, Brazil; McGuire Center for Lepidoptera and Biodiversity, Florida Museum of Natural History, University of Florida, Gainesville, USA; Mike J. Perceval collection, Surrey, UK; Museo de Historia Natural, Universidad Nacional Mayor de San Marcos, Lima, Peru; Pontificia Universidad Católica del Ecuador, Quito, Ecuador; Zoologische Staatssammlung München, Munich, Germany; Museu de Zoologia ’Adão José Cardoso’ da Universidade Estadual de Campinas, Campinas, Brazil. All specimens housed at the McGuire Center for Lepidoptera and Biodiversity and Museo de Historia Natural are databased and accession numbers are: MUSM-LEP 103506, MUSM-LEP 103534, MUSM-LEP 103511, MUSM-LEP 103513, MUSM-LEP 103514, MUSM-LEP 103514, MUSM-LEP 103519, MUSM-LEP 103513, MUSM-LEP 103504, MUSM-LEP 103519, MUSM-LEP 103544, MUSM-LEP 103537, MUSM-LEP 103543, FLMNH-MGCL-1036473, FLMNH-MGCL-1036467, FLMNH-MGCL-1036470, FLMNH-MGCL-1036468, FLMNH-MGCL-1036472, FLMNH-MGCL-1036465, FLMNH-MGCL-1036466, FLMNH-MGCL-281451, FLMNH-MGCL-195446, FLMNH-MGCL-288755, FLMNH-MGCL-288757, FLMNH-MGCL-288756, FLMNH-MGCL-209874, FLMNH-MGCL-209869, FLMNH-MGCL-195182, FLMNH-MGCL-195186, FLMNH-MGCL-195183. FLMNH-MGCL-195184, FLMNH-MGCL-195185, FLMNH-MGCL-195187, FLMNH-MGCL-297421, FLMNH-MGCL-297422, FLMNH-MGCL-145666, FLMNH-MGCL-150973, FLMNH-MGCL-209871, FLMNH-MGCL422 1036471, FLMNH-MGCL433 1036469.

### New Species Registration

The following information was supplied regarding the registration of a newly described species: Publication LSID: urn:lsid:zoobank.org:pub:DC9E9E4F-0822-4A0A-BAFB-16F078089C47. *Scriptor* Nakahara & Espeland, n. gen. LSID: urn:lsid:zoobank.org:act:736AB6F3-6BF3-433E-AA1B-E6B06AE6C8F8. *Scriptor sphenophorus* n. gen. and **n. sp.** LSID: urn:lsid:zoobank.org:act:D55DC37F-BFF1-45E2-AE36-3485EA75A104.
